# Bioinformatics Analysis of Expression Profiles and Prognostic Values of the Signal Transducer and Activator of Transcription Family Genes in Glioma

**DOI:** 10.3389/fgene.2021.625234

**Published:** 2021-07-02

**Authors:** Wei Ji, Yuankun Liu, Bin Xu, Jie Mei, Chao Cheng, Yong Xiao, Kun Yang, Weiyi Huang, Jiantong Jiao, Hongyi Liu, Junfei Shao

**Affiliations:** ^1^Department of Neurosurgery, Wuxi People’s Hospital of Nanjing Medical University, Wuxi, China; ^2^Department of Neurosurgery, Nanjing Brain Hospital Affiliated to Nanjing Medical University, Nanjing, China

**Keywords:** glioma, signal transducer and activator of transcription, prognosis, The Cancer Genome Atlas, Chinese Glioma Genome Atlas

## Abstract

Signal transducer and activator of transcription (*STAT*) family genes—of which there are seven members: *STAT1, STAT2, STAT3, STAT4, STAT5A, STAT5B*, and *STAT6*—have been associated with the progression of multiple cancers. However, their prognostic values in glioma remain unclear. In this study, we systematically investigated the expression, the prognostic value, and the potential mechanism of the *STAT* family genes in glioma. The expression of *STAT*1/2/3/5A/6 members were significantly higher and positively correlated with *IDH* mutations, while the expression of *STAT5B* was lower and negatively correlated with *IDH* mutations in glioma. Survival analysis indicated that the upregulation of *STAT1/2/3/5A/6* and downregulation of *STAT5B* expression was associated with poorer overall survival in glioma. Joint effects analysis of *STAT1/2/3/5A/5B/6* expression suggested that the prognostic value of the group was more significant than that of each individual gene. Thus, we constructed a risk score model to predict the prognosis of glioma. The receiver operating characteristic curve and calibration curves showed good performance as prognostic indicators in both TCGA (The Cancer Genome Atlas) and the CGGA (Chinese Glioma Genome Atlas) databases. Furthermore, we analyzed the correlation between *STAT* expression with immune infiltration in glioma. The Protein–protein interaction network and enrichment analysis showed that *STAT* members and co-expressed genes mainly participated in signal transduction activity, Hepatitis B, the Jak-STAT signaling pathway, transcription factor activity, sequence-specific DNA binding, and the cytokine-mediated signaling pathway in glioma. In summary, our study analyzed the expression, prognostic values, and biological roles of the *STAT* gene family members in glioma, based on which we developed a new risk score model to predict the prognosis of glioma more precisely.

## Introduction

Glioma is one of the most common cancers and leading causes of cancer-related deaths worldwide (Gagliardi et al., 2014; Zhang and Zhang, 2015). Despite the great improvement in therapy, the overall survival of glioma patients remains poor (Linz, 2010). Thus, it is essential to reveal the molecular mechanism involved in tumorigenesis and to identify novel prognosis biomarkers and therapeutic targets for glioma.

The Signal transducer and activator of transcription (*STAT*) gene family contains seven members: *STAT1*, *STAT2*, *STAT3*, *STAT4*, *STAT5A*, *STAT5B*, and *STAT6* (Gao et al., 2012). The STAT proteins are large proteins (750–850 amino-acids in size) and all share five common domains: an amino-terminal domain, a coiled-coil domain, a DNA-binding domain (DBD), an SRC homology 2 (SH2) domain, and a transactivation domain (TAD) (Lorenzini et al., 2017). Among them, the coiled-coil domain contributes to nuclear localization; the DBD domain is relevant to target gene transcription; the SH2 domain mediates homo- or heterodimerization of STATs; and the TAD domain is crucial for the activity of the STAT protein (Miklossy et al., 2013; Dorritie et al., 2014). STAT proteins are regarded as cytoplasmic transcription factors that regulate the processes of cell proliferation, differentiation, apoptosis, and immune responses by modulating the transcription of target genes (Goswami and Kaplan, 2017; Villarino et al., 2017). In recent years, an increasing number of studies have demonstrated that constitutive activation of *STATs* participates in the pathology of glioma. Studies have also shown that the expression of *STAT1* is decreased in glioma compared with normal brain tissues (Ju et al., 2013; Chen et al., 2020a). Overexpression of STAT1 strongly suppresses the growth of glioma cells and promotes cell apoptosis (Zhang et al., 2018). Ehrmann et al. (2008) reported that the expression of STAT2 was lower in low-grade astrocytomas than in high-grade astrocytomas, while the function of STAT2 in glioma has rarely been reported. STAT3 has been discovered to be frequently activated and has been identified as an oncogene in glioma. Constitutively activated STAT3 in glioma is associated with oncogenesis and cancer progression (de la Iglesia et al., 2009; Chai et al., 2016). However, recent studies have also shown that STAT3 may play a tumor suppressive function in glioma pathogenesis when associated with different levels of expression of *PTEN* (de la Iglesia et al., 2008).

Studies investigating the expression and function of the *STAT4* gene in glioma are rare. STAT5 comprises two highly homologous isoforms, STAT5A (94 kDa) and STAT5B (92 kDa) (Trifa et al., 2013), which share considerable functional overlap but play different roles (Socolovsky et al., 1999). The expression of STAT5A showed no significant correlation with high grade glioma when compared to normal brain tissues and low-grade glioma, although the knockdown of STAT5A expression inhibited cell invasion but not cell growth in glioblastoma (GBM) (Liang et al., 2009). *STAT5B* was recognized as the pertinent gene contributing to the progression from low-grade glioma (LGG) to HGG. Silencing *STAT5B* inhibited cell growth and cell invasion in the GBM cell line (Liang et al., 2005). Higher STAT6 levels were found in glioma tissues than normal brain tissues. Importantly, knockdown of *STAT6* expression in a GBM cell line decreased cell proliferation and invasion (Merk et al., 2011).

Collectively, these studies suggest that the *STAT* gene family plays an important role in the development and progression of glioma. However, the potential application of the entire *STAT* gene family remains unclear. In this study, the predictive value, potential mechanism, and correlation with tumor-immune infiltrating cells (TIICs) of the *STAT* gene family were comprehensively assessed using bioinformatics tools.

## Materials and Methods

### Expression of the *STAT* Gene Family in Public Databases

The Oncomine database^1^ was first used to analyze the mRNA expression of the *STAT* gene family in different types of cancers in comparison with normal tissues (threshold *p*-Value = 0.01 and threshold fold change = 2) (Nie et al., 2020). Second, TCGA^2^ and CGGA^3^ databases were included to analyze the expression and Spearman’s correlations of *STAT* gene family across different tumor grades (WHO grade II–IV) and different *IDH* mutation statuses (mutant and wild-type) of glioma samples (Liu et al., 2020). Next, the protein expression levels of the *STAT* gene family in normal brain tissues and glioma tissues were explored in the Human Protein Atlas^4^ (Uhlen et al., 2010). Finally, the genomic alterations of the *STAT* gene family were explored using the cBioPortal^5^.

### Prognostic Value of the *STAT* Gene Family in Glioma

The prognostic values of each member of the *STAT* family were evaluated in TCGA and the CGGA databases. Then, Least absolute shrinkage and selection operator (LASSO) regression analysis was performed to construct a risk score model based on the expression and prognostic value of *STAT* gene family members in the TCGA dataset. Cases extracted from the TCGA and CGGA datasets were stratified into high- and low-risk groups based on the risk score. The time-dependent receiver operating characteristic (ROC) curve was drawn to evaluate the predictive value of this risk score for overall survival in both datasets.

### Correlation Analysis Between *STAT* Gene Family Members and Tumor-Immune Infiltrating Cells

The association between the *STAT* gene family members and all TIICs (including B-cells, CD8 + T-cells, CD4 + T-cells, macrophages, neutrophils, and dendritic cells) in LGG and GBM were analyzed using the Tumor Immune Estimation Resource (TIMER) platform^6^. Tumor purity was used to correct the Spearman-based correlation analysis (Li et al., 2017).

### GeneMANIA Analysis

The GeneMANIA website was used to predict function and to construct protein-protein interaction (PPI) networks of genes or gene lists through bioinformatics methods (Warde-Farley et al., 2010). Co-expression, co-location, physical interaction, and gene enrichment were analyzed using this web-based interface.

### Functional and Pathway Enrichment Analysis

The interactive genes of the *STAT* gene family, identified from GeneMANIA, were subject to Gene Ontology (GO) and Kyoto Encyclopedia of Genes and Genomes (KEGG) enrichment analysis using Database for Annotation, Visualization, and Integrated Discovery (DAVID) (Huang et al., 2007). A *p*-Value < 0.05 was considered statistically significant.

### Statistical Analysis

Graphpad Prism version 5.0 software (GraphPad Software, San Diego, CA, United States) and IBM SPSS Statistics version 20.0 (Chicago, IL, United States) were used for statistical analysis and graphing. Cox regression analysis was employed to perform univariate and multivariate survival analysis. The student’s *t*-test and Pearson’s correlation test were conducted to compare groups and for correlation analysis. A *p*-Value of <0.05 was considered statistically significant in our study.

## Results

### Expression of the *STAT* Gene Family in Glioma

**FIGURE 1:**
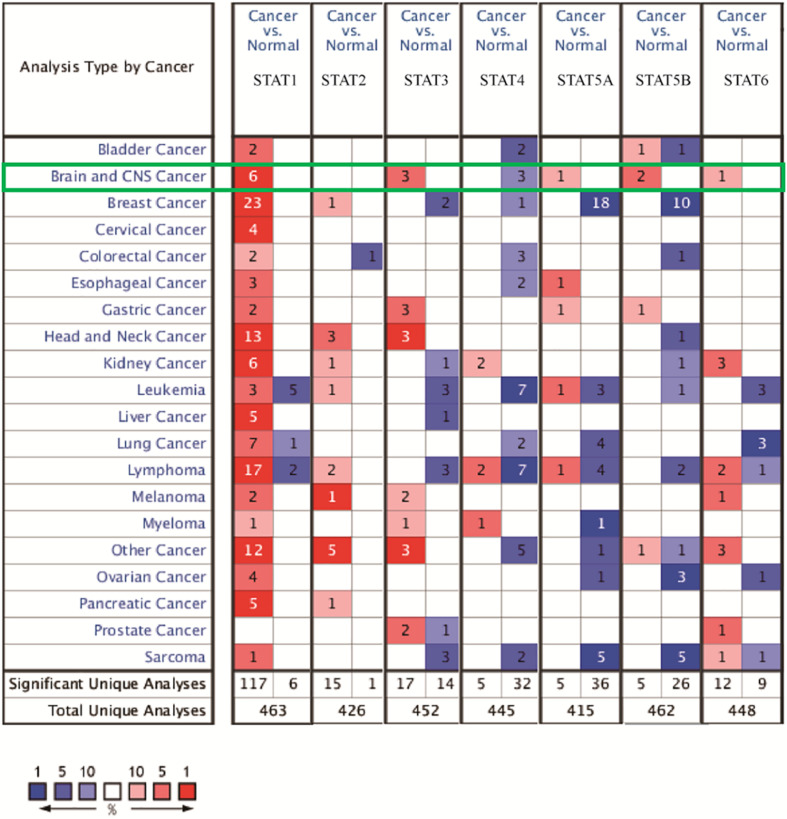
mRNA expression of the *STAT* gene family in different types of cancers (analyzed with Oncomine database). Search parameters were: fold change = 2, *P*-value = 0.01. The value in the tables represents the number of datasets that conform to the thresholds. The color intensity (red or blue) is positively related to the degree of upregulation or downregulation, respectively. The mRNA expression of the *STAT* gene family in glioma is sketched with green highlights.

**FIGURE 2:**
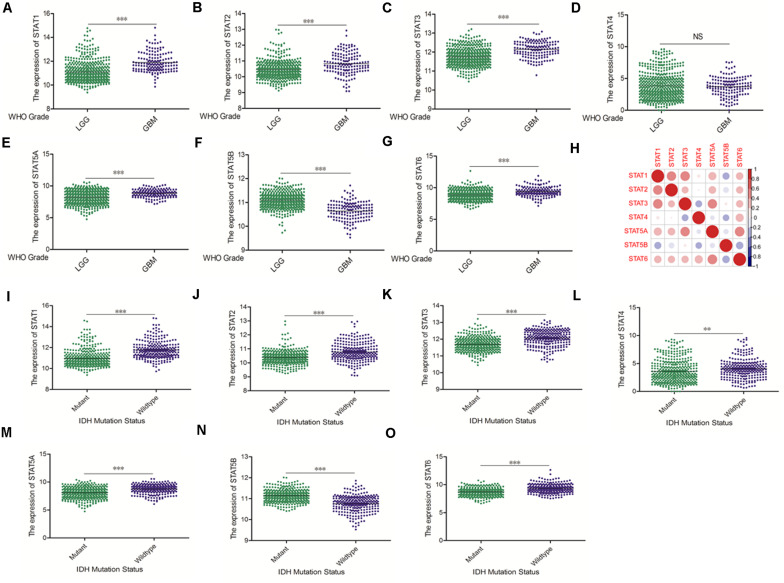
mRNA expression levels of *STATs* in glioma (using TCGA database). **(A–G)** mRNA expression levels of *STAT* genes in LGG and GBM (^∗∗^*p* < 0.01 and ^∗∗∗^*p* < 0.001). **(H)** Co-expression heat map of *STAT* genes in glioma. **(I–O)** Correlation between *STAT* genes expression and *IDH* mutation status in glioma (^∗∗^*p* < 0.01 and ^∗∗∗^*p* < 0.001). “NS” means “not significant” or “not statistically significant,” i.e., *p* ≥ 0.05.

**FIGURE 3:**
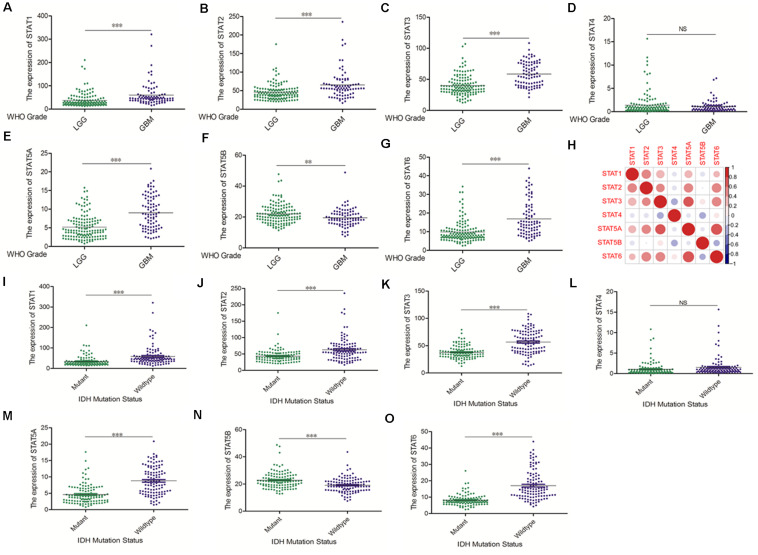
mRNA expression levels of *STATs* in glioma (CGGA). **(A–G)** mRNA expression levels of *STAT* genes in LGG and GBM using CGGA database (^∗∗^*p* < 0.01, and ^∗∗∗^*p* < 0.001). **(H)** Co-expression heat map of *STAT* genes in glioma using CGGA database. **(I–O)** The correlation between *STAT* gene expression and *IDH* mutation status in glioma using CGGA database (^∗∗^*p* < 0.01 and ^∗∗∗^*p* < 0.001). “NS” means “not significant” or “not statistically significant,” i.e., *p* ≥ 0.05.

**FIGURE 4:**
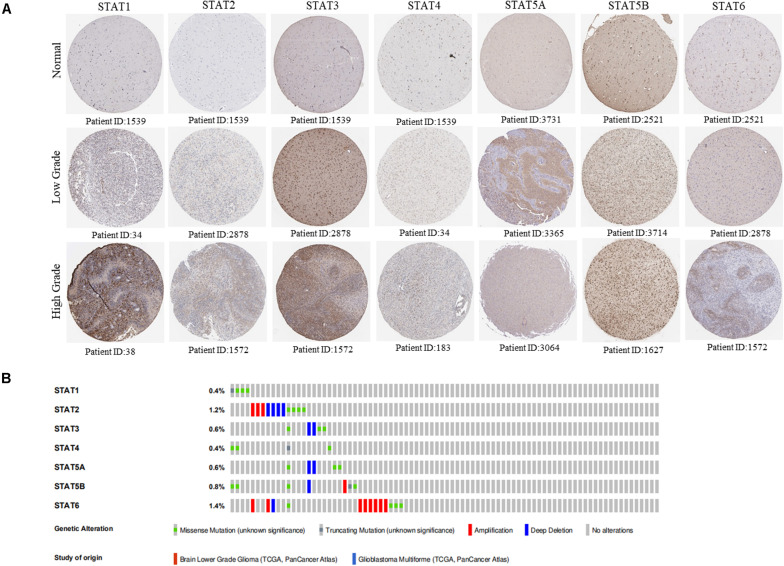
Protein expression level and genetic alteration of *STATs* in glioma**. (A)** The protein expression of *STAT* genes in glioma (low grade and high grade) and normal brain tissues using human protein atlas database. **(B)** Genetic variations of *STAT* genes in glioma using OncoPrint.

The Oncomine database was employed to analyze the mRNA expression of members of the *STAT* gene family in various types of cancers (Figure 1 and Table 1). *STAT1*, *STAT3*, *STAT5A*, *STAT5B*, and *STAT6* mRNA expression levels were significantly upregulated in glioma (including GBM, astrocytoma, oligodendroglioma, and anaplastic astrocytoma), compared with normal brain tissues or neural stem cells. However, the expression level of *STAT4* mRNA was lower in glioma than in normal controls. No study has been conducted to evaluate *STAT2* mRNA in the Oncomine database. Next, TCGA datasets were used to evaluate the expression of *STAT* gene family members in glioma. The results showed that the expression of *STAT1*, *STAT2*, *STAT3*, *STAT5A*, and *STAT6* increased with increasing tumor grades. While the expression of *STAT5B* was negatively correlated with tumor grades. mRNA expression of *STAT4* showed no statistically significant differences between LGG (low-grade glioma) and GBM (Figures 2A–G). Spearman’s correlations of the expression of each member in the *STAT* gene family in the TCGA dataset were also analyzed (Figure 2H). Furthermore, the mRNA expression of *STAT1*, *STAT2*, *STAT3*, *STAT4*, *STAT5A*, and *STAT6* were upregulated in *IDH* wild-type compared to *IDH* mutated cases, but *STAT5B* showed the opposite results (Figures 2I–O). Similar results were also obtained in the CGGA dataset, except that the mRNA expression of *STAT4* showed no significant correlation with the IDH-mutational status (Figures 3A–O). The association between *STAT* expression and clinicopathological characteristics was also performed (Supplementary Tables 1, 2). The results indicated that *STAT* expression was correlated with tumor grades, *IDH* genotype, and age. The protein levels of *STAT* gene family members in normal brain tissues and glioma samples (low-grade and high-grade) were explored using the Human Protein Atlas website (Figure 4A). Finally, the genomic alterations of the *STAT* gene family in glioma were investigated through the cBioPortal website. As shown in Figure 4B, *STAT6* displayed the highest incidence rate of genetic variations (1.4%), which was followed by *STAT2* (1.2%), *STAT5*B (0.8%), *STAT2* (0.6%), *STAT5A* (0.6%), *STAT1* (0.4%), and *STAT4* (0.4%).

**TABLE 1:** Datasets of *STAT* family genes in glioma (Oncomine).

Gene	Tumor (cases)	Normal (cases)	Fold change	*t*-test	*P*-value	Dataset
*STAT1*	Glioblastoma (30)	Brain (2) and Cerebellum (1)	3.136	9.074	1.58E-10	Liang et al.
	Atypical Teratoid/Rhabdoid Tumor (5)	Cerebellum (4)	3.989	3.866	0.003	Pomeroy et al.
	Glioblastoma (542)	Brain (10)	2.085	13.870	1.49E-11	TCGA
	Glioblastoma (27)	White Matter (7)	2.701	6.363	1.69E-5	Shai et al.
	Astrocytoma (5)	White Matter (7)	2.094	4.391	6.77E-4	Shai et al.
	Oligodendroglioma (3)	White Matter (7)	2.129	5.585	4.16E-4	Shai et al.
*STAT2*	–	–	–	–	–	–
*STAT3*	Glioblastoma (27)	Brain (4)	2.076	11.595	2.81E-7	Bredel et al.
	Glioblastoma (542)	Brain (10)	2.226	13.114	5.83E-8	TCGA
	Glioblastoma (81)	Brain (23)	2.270	8.047	2.30E-10	Sun et al.
*STAT4*	Glioblastoma (542)	Brain (10)	−7.704	−21.720	6.12E-10	TCGA
	Anaplastic Astrocytoma (19)	Brain (23)	−2.510	−6.083	2.77E-7	Sun et al.
	Oligodendroglioma (50)	Brain (23)	−2.369	−6.909	2.58E-9	Sun et al.
*STAT5A*	Glioblastoma (22)	Neural Stem Cell (3)	2.255	7.909	7.46E-4	Lee et al.
*STAT5B*	Oligodendroglioma (50)	Brain (23)	3.014	6.927	5.66E-9	Sun et al.
	Glioblastoma (81)	Brain (23)	2.953	7.559	4.61E-9	Sun et al.
*STAT6*	Glioblastoma (22)	Neural Stem Cell (3)	2.103	4.798	0.004	Lee et al.

*TCGA, The Cancer Genome Atlas.*

### Prognostic Potential of *STAT* Gene Family in Glioma

**FIGURE 5:**
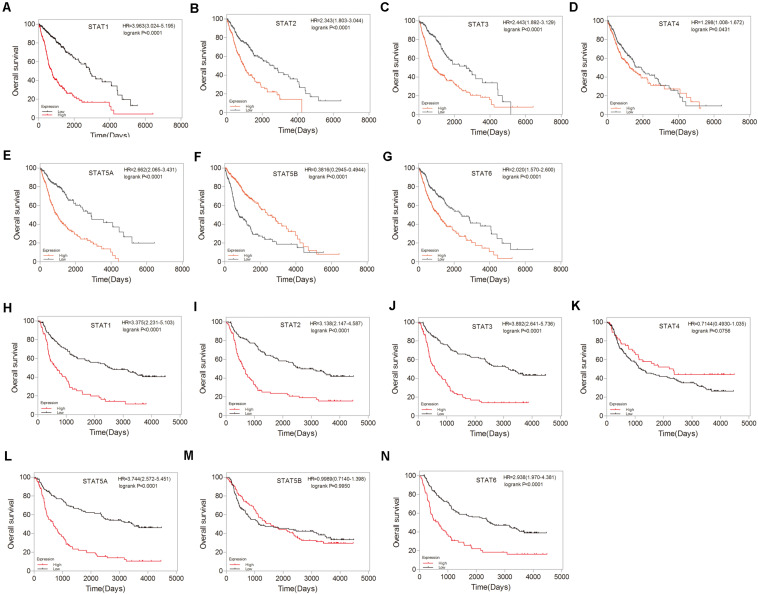
Prognostic values of *STATs* in glioma. **(A–G)** Prognostic significance of individual *STAT* genes in glioma using TCGA database. **(H–N)** Survival curves of individual *STAT* genes in glioma using CGGA database.

To investigate the prognostic value of the *STAT* gene family in glioma, survival analysis was performed to determine the correlation between the expression of *STAT* gene family members and the prognosis of glioma patients. High mRNA expression of *STAT1*, *STAT2*, *STAT3*, *STAT5A*, and *STAT6* indicated poorer prognosis in both TCGA and CGGA datasets (*p* < 0.0001) (Figures 5A–C,E,G–J,L,N). No apparent correlations between the mRNA expression of *STAT4* and the prognosis of glioma were found in either the TCGA or CGGA datasets (Figures 5D,K). Inconsistent results were obtained from the TCGA data (hazard ratio [HR] = 0.3816, 95% CI = 0.2945–0.4944, *p* < 0.0001) (Figure 5F) and CGGA (HR = 0.9989, 95%CI = 0.7140–1.398, *p* = 0.9950) (Figure 5M) when analyzing the relationship between STAT5B and the prognosis of glioma.

### Construction of a Prognostic Gene Signature Based on *STAT* Gene Family Members

**FIGURE 6:**
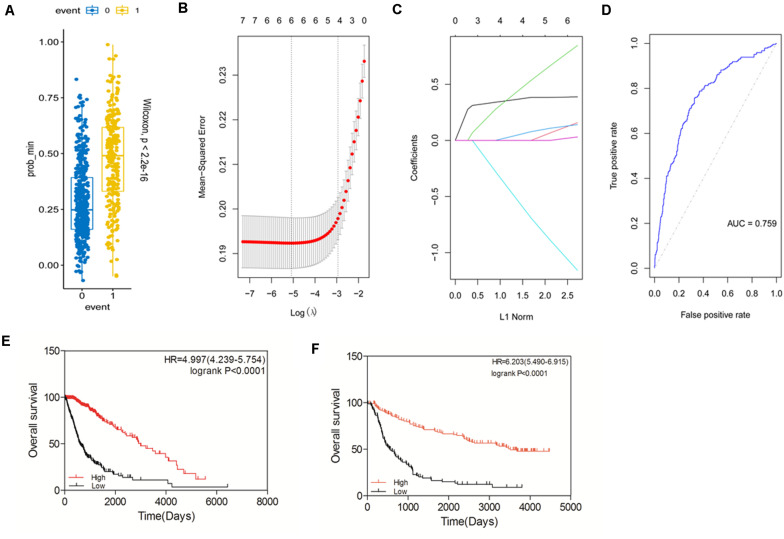
Construction of a prognostic gene signature model based on the *STAT* gene family. (**A–D)** Construction of a new prognostic signature. **(E)** Prognostic value of the new signature in glioma using TCGA database. **(F)** Prognostic value of the new signature in glioma using CGGA database.

Based on the expression and prognostic value of the *STAT* gene family in glioma derived from the TCGA dataset, we aimed to construct a new prognostic gene signature that could predict the prognosis of glioma more accurately (Figures 6A–D). The risk score of the model was calculated as follows: expression of *STAT1* × 0.082732073 + expression of *STAT2* × 0.038846769 + expression of *STAT3* × 0.175344356 + expression of *STAT5A* × 0.025186483- expression of *STAT5B* × 0.235800802 + expression of *STAT6* × 0.007629574. The area under the ROC curve (AUC) was 0.759. The prognostic value of this model was tested in the TCGA dataset. We found that high-risk patients had a significantly poorer OS than the low-risk group (*p* < 0.0001) (Figure 6E). The HR was 4.997 (4.239–5.747), which was higher than the other patient groups in Figures 5A–G. Similar results were obtained using the CGGA dataset (Figure 6F). The above findings indicated that the newly constructed model had good performance for glioma survival prediction.

To eliminate the influence of tumor grades and *IDH* type, we assessed the prognostic value of STATs in LGG/GBM and *IDH* wild-type/mutated, respectively. The result showed that *STAT1/2/3/5A/6* and the new model were significantly associated with the prognosis in LGG in both TCGA and CGGA datasets. However, none of the STATs showed a significant correlation with the prognosis in the GBM group of either dataset. Only our new model showed a weak correlation with the prognosis of GBM in the CGGA dataset (*p* = 0.0278). In the TCGA dataset, the expression of members and our constructed model was significantly correlated with prognosis both in *IDH* wild-type and *IDH*-mutated cases. In the CGGA dataset, only our constructed model was significantly correlated with prognosis in both the *IDH*-wild type and *IDH*-mutated cases (Tables 2, 3). These findings indicated that our newly constructed model showed good performance in subgroup analysis.

**TABLE 2:** Subgroup analysis of the prognostic value of STATs in TCGA.

Gene	Variable	LGG	GBM	IDH-wild	IDH-mut
*STAT1*	HR	0.3411	0.9603	0.6173	0.5378
	95%CI	0.2366-0.4917	0.6608-1.396	0.4502-0.8464	0.3369-0.8586
	*P*-value	<0.0001	0.8320	0.0027	0.0094
*STAT2*	HR	0.5420	0.8141	0.7065	0.7648
	95%CI	0.3708-0.7923	0.5634-1.176	0.5186-0.9625	0.4685-1.249
	*P*-value	0.0016	0.2734	0.0276	0.2837
*STAT3*	HR	0.5147	0.7864	0.5498	0.6344
	95%CI	0.3609-0.7341	0.5441-1.137	0.4030-0.7500	0.4013-1.003
	*P*-value	0.0002	0.2009	0.0002	0.0514
*STAT4*	HR	0.7268	0.9123	1.052	1.206
	95%CI	0.5075-1.041	0.6303-1.320	0.7709-1.435	0.7608-1.913
	*P*-value	0.0816	0.6265	0.7508	0.4251
*STAT5A*	HR	0.4604	0.8298	0.6792	0.6681
	95%CI	0.3222-0.6580	0.5745-1.199	0.4991-0.9243	0.4183-1.067
	*P*-value	<0.0001	0.3200	0.0139	0.0913
*STAT5B*	HR	1.241	0.9398	1.191	0.7730
	95%CI	0.8696-1.770	0.6514-1.356	0.8726-1.625	0.4907-1.218
	*P*-value	0.2343	0.7398	0.2712	0.2667
*STAT6*	HR	0.6306	0.8302	0.7144	0.5877
	95%CI	0.4405-0.9029	0.5735-1.202	0.5210-0.9796	0.3716-0.9293
	*P*-value	0.0118	0.3240	0.0368	0.0230
The new model	HR	0.3723	0.9060	0.5307	0.5782
	95%CI	0.2593-0.5345	0.6275-1.308	0.3886-0.7247	0.3663-0.9128
	*P*-value	<0.0001	0.5983	<0.0001	0.0187

*HR, Hazard Ratio; CI, confidence interval.*

**TABLE 3:** Subgroup analysis of the prognostic value of STATs in CGGA.

Gene	Variable	LGG	GBM	IDH-wild	IDH-mut
*STAT1*	HR	0.2969	0.9817	0.7434	0.3343
	95%CI	0.1616-0.5457	0.5740 −1.679	0.4548 −1.215	0.1614-0.6923
	*P*-value	<0.0001	0.9464	0.2369	0.0032
*STAT2*	HR	0.4099	0.6458	0.5286	0.6426
	95%CI	0.2446-0.6870	0.3740-1.115	0.3284-0.8507	0.3613-1.143
	*P*-value	0.0007	0.1166	0.0449	0.1323
*STAT3*	HR	0.3223	0.6319	0.6328	0.6385
	95%CI	0.1855-0.5602	0.3912-1.021	0.4128-0.9702	0.3562-1.145
	*P*-value	<0.0001	0.0606	0.033	0.1319
*STAT4*	HR	1.743	1.185	2.073	1.751
	95%CI	0.9993-3.042	0.7242-1.940	1.307-3.289	0.9624-3.186
	*P*-value	0.0503	0.4989	0.002	0.0666
*STAT5A*	HR	0.2426	0.8031	0.7144	0.3887
	95%CI	0.1415-0.4157	0.5017-1.286	0.4646-1.099	0.2116-0.7140
	*P*-value	<0.0001	0.3611	0.1257	0.0023
*STAT5B*	HR	0.7189	1.197	0.9728	0.5065
	95%CI	0.4374-1.181	0.7522-1.905	0.6393-1.480	0.2819-0.9100
	*P*-value	0.1928	0.4479	0.8976	0.0229
*STAT6*	HR	0.5064	0.6184	0.7195	0.577
	95%CI	0.2937-0.8729	0.3730-1.025	0.4613-1.122	0.3198-1.041
	*P*-value	0.0143	0.0624	0.1468	0.0679
The new model	HR	0.2594	0.5756	0.5161	0.5663
	95%CI	0.1442-0.4666	0.3519-0.9416	0.3328-0.8004	0.3017-1.063
	*P*-value	<0.0001	0.0278	0.0031	0.0767

*HR, Hazard Ratio; CI, confidence interval.*

### Relevance of *STAT* Gene Family and TIICs in Glioma

**FIGURE 7:**
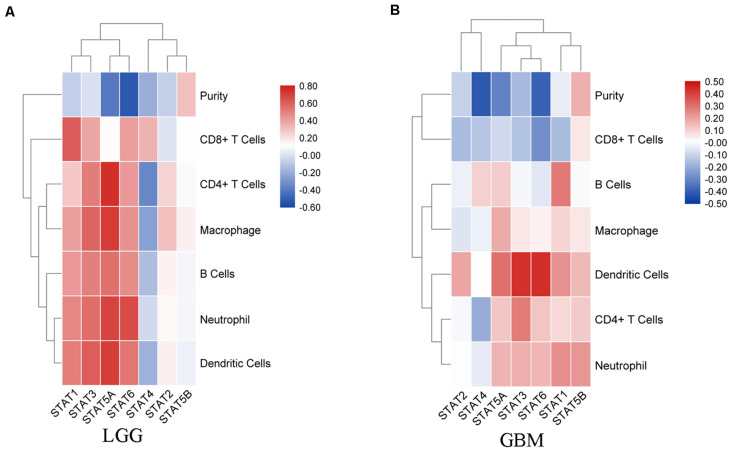
Correlation analysis between *STAT* genes and TIICs. **(A)** Correlation between *STATs* and each type of TIICs (B-cells, CD4 + T-cells, CD8 + T-cells, neutrophils, macrophages, and dendritic cells) in LGG; **(B)** Correlation between *STATs* and each type of TIICs (B-cells, CD4 + T-cells, CD8 + T-cells, neutrophils, macrophages, and dendritic cells) in GBM.

In recent years, immunotherapy has become a new hope for improving the prognosis of glioma. The importance of the immune infiltrate has attracted an increasing amount of attention. Thus, the correlation between TIICs and the STAT gene family was explored using the TIMER website. In LGG, the expression of STAT1 was positively associated with B cells (Cor = 0.426, *p* = 1.82e-22), CD8 + T cells (Cor = 0.615, *p* = 4.22e-51), CD4 + T cells (Cor = 0.282, *p* = 3.65e-10), macrophages (Cor = 0.413, *p* = 6.07e-21), neutrophils (Cor = 0.484, *p* = 2.19e-29), and dendritic cells (Cor = 0.512, *p* = 3.40e-33). *STAT2, STAT3, STAT5A*, and *STAT6* expression showed similar results. The expression of *STAT5B* was positively associated with the infiltration abundance of CD8 + T cells (Cor = 0.116, *p* = 1.13e-02), CD4 + T cells (Cor = 0.096, *p* = 3.59e-02), and macrophages (Cor = 0.159, *p* = 5.36e-04). *STAT4* was negatively associated with B cells (Cor = −0.14, *p* = 2.12e-03), CD4 + T cells (Cor = −0.346, *p* = 7.98e-15), macrophages (Cor = −0.247, *p* = 5.59e-08), and dendritic cells (Cor = −0.185, *p* = 5.10e-05), and was positively associated with CD8 + T cells (Cor = 0.35, *p* = 3.26e-15) (Figure 7A). In GBM, the association between STATs and TICCs was very different. *STAT1* was positively associated with B cells, macrophages, neutrophils, and dendritic cells, but was negatively associated with CD8 + T cells (Cor = −0.159, *p* = 1.09e-03). *STAT2* was positively associated with dendritic cells (Cor = 0.195, *p* = 5.81e-05) and negatively associated with CD8 + T cells (Cor = −0.161, *p* = 9.46e-04). STAT3 was negatively associated with CD8 + T cells (Cor = −0.146, *p* = 2.85e-03) and was positively correlated with CD4 + T cells, neutrophils, and dendritic cells. *STAT4* was negatively associated with CD8 + T cells and CD4 + T cells and positively associated with B cells (Cor = 0.107, *p* = 2.91e-02). *STAT5A* was positively associated with B cells (Cor = 0.115, *p* = 1.91e-02), CD4 + T cells (Cor = 0.127, *p* = 9.55e-03), macrophages (Cor = 0.183, *p* = 1.64e-04), neutrophils (Cor = 0.166, *p* = 6.63e-04) and dendritic cells (Cor = 0.306, *p* = 1.60e-10). *STAT5B* was positively associated with CD4 + T cells (Cor = 0.119, *p* = 1.47e-02), neutrophils (Cor = 0.226, *p* = 3.06e-06), and dendritic cells (Cor = 0.155, *p* = 1.45e-03). *STAT6* was negatively associated with CD8 + T cells and positively with CD4 + T cells, neutrophils and dendritic cells (Figure 7B).

### Predicted Pathways of the *STAT* Gene Family in Glioma

**FIGURE 8:**
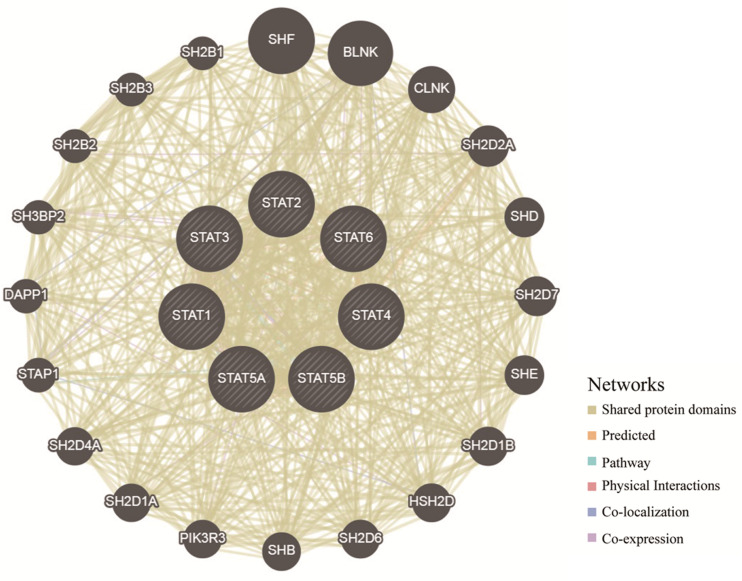
Protein–protein interaction network of *STAT* family members (GeneMANIA). The colors of the lines between different genes represent the methods performed: Shared protein domains, Predicted, Pathway, Physical Interactions, Co-localization, and Co-expression.

To explore the potential mechanisms through which the *STAT* gene family participates in the pathology of glioma, GeneMANIA was used to predict genes co-expressed with the *STAT* gene family and to construct a PPI network. A total of 20 genes were found to be co-expressed with the *STAT* gene family (Figure 8). Next, these genes were subjected to GO and KEEG analyses using the DAVID database. The top 5 molecular functions (MF) enriched terms were signal transducer activity, transcription factor activity, and sequence-specific DNA binding, signaling adaptor activity, DNA binding, and SH3/SH2 adaptor activity. The top 5 biological processes (BP) enriched terms with which the *STAT* gene family and their cooperators were significantly associated were: the cytokine-mediated signaling pathway; the Jak-STAT cascade; intracellular signal transduction; positive regulation of mitotic cell cycle; and transcription and DNA-templates. The top two cellular components enriched terms were cytoplasm and nuclear chromatin (Table 4). The top 10 KEGG enriched terms were Hepatitis B, the Jak-STAT signaling pathway, measles, the prolactin signaling pathway, acute myeloid leukemia, inflammatory bowel disease (IBD), the chemokine signaling pathway, natural killer cell-mediated cytotoxicity, the neurotrophin signaling pathway, and hepatitis C (Table 5).

**TABLE 4:** Gene ontology terms enrichment (including TOP 5 MF, TOP 5 BP and TOP 2 CC).

Category	Term2	Count	*P*-value	Genes	Fold Enrichment	FDR
GOTERM_MF_DIRECT	GO:0004871∼signal transducer activity	10	1.56E-15	*STAT6, STAT4, STAT5A, STAT5B, SH2B3, SH2B2, SH2B1, STAT1, STAT3, STAT2*	66.13636	1.20E-12
GOTERM_MF_DIRECT	GO:0003700∼transcription factor activity, sequence-specific DNA binding	7	4.19E-06	*STAT6, STAT4, STAT5A, STAT5B, STAT1, STAT3, STAT2*	13.34279	0.003235
GOTERM_MF_DIRECT	GO:0035591∼signaling adaptor activity	3	2.96E-05	*SH2B3, SH2B2, SH2B1*	327.375	0.022862
GOTERM_MF_DIRECT	GO:0003677∼DNA binding	6	3.80E-04	*STAT6, STAT5A, STAT5B, STAT1, STAT3, STAT2*	8.184375	0.293076
GOTERM_MF_DIRECT	GO:0005070∼SH3/SH2 adaptor activity	2	0.005335	*SH2D1A, BLNK*	349.2	4.046197
GOTERM_BP_DIRECT	GO:0019221∼cytokine-mediated signaling pathway	5	1.23E-05	*STAT4, STAT5A, STAT5B, SH2B2, STAT1*	31.91136	0.014609
GOTERM_BP_DIRECT	GO:0007259∼JAK-STAT cascade	3	1.24E-04	*STAT5A, STAT5B, STAT1*	167.9028	0.14728
GOTERM_BP_DIRECT	GO:0035556∼intracellular signal transduction	5	3.81E-04	*SH2B3, SH2B2, CLNK, SH2B1, BLNK*	13.18078	0.452966
GOTERM_BP_DIRECT	GO:0045931∼positive regulation of mitotic cell cycle	3	4.72E-04	*SHB, STAT5A, STAT5B*	87.30947	0.560178
GOTERM_BP_DIRECT	GO:0006351∼transcription, DNA-templated	6	8.29E-04	*STAT6, STAT5A, STAT5B, STAT1, STAT3, STAT2*	7.239592	0.981594
GOTERM_CC_DIRECT	GO:0005737∼cytoplasm	9	0.01126	*SH2D4A, STAT6, SH2D1A, STAT5A, STAT5B, STAT1, STAT3, STAT2, BLNK*	2.486491	7.726748
GOTERM_CC_DIRECT	GO:0000790∼nuclear chromatin	3	0.011558	*STAT6, STAT1, STAT3*	17.17889	7.923914

*MF, molecular functions; BP, biological processes, CC, cellular component.*

**TABLE 5:** Top 10 of KEGG pathway enrichment.

Category	Term2	Count	*P*-Value	Genes	Fold Enrichment	FDR
KEGG_PATHWAY	bta05161:Hepatitis B	8	9.53E-09	*STAT6, STAT4, STAT5A, STAT5B, PIK3R3, STAT1, STAT3, STAT2*	24.00636	1.00E-05
KEGG_PATHWAY	bta04630:Jak-STAT signaling pathway	8	1.10E-08	*STAT6, STAT4, STAT5A, STAT5B, PIK3R3, STAT1, STAT3, STAT2*	23.52941	1.15E-05
KEGG_PATHWAY	bta05162:Measles	7	2.51E-07	*SH2D1A, STAT5A, STAT5B, PIK3R3, STAT1, STAT3, STAT2*	22.20588	2.63E-04
KEGG_PATHWAY	bta04917:Prolactin signaling pathway	5	1.42E-05	*STAT5A, STAT5B, PIK3R3, STAT1, STAT3*	30.00795	0.014858
KEGG_PATHWAY	bta05221:Acute myeloid leukemia	4	2.02E-04	*STAT5A, STAT5B, PIK3R3, STAT3*	31.72269	0.211964
KEGG_PATHWAY	bta05321:Inflammatory bowel disease (IBD)	4	3.92E-04	*STAT6, STAT4, STAT1, STAT3*	25.37815	0.410689
KEGG_PATHWAY	bta04062:Chemokine signaling pathway	5	5.04E-04	*STAT5B, PIK3R3, STAT1, STAT3, STAT2*	12.00318	0.527852
KEGG_PATHWAY	bta04650:Natural killer cell mediated cytotoxicity	4	0.001753	*SH2D1A, PIK3R3, SH2D1B, SH3BP2*	15.18351	1.824594
KEGG_PATHWAY	bta04722:Neurotrophin signaling pathway	4	0.002119	*SH2B3, SH2B2, SH2B1, PIK3R3*	14.21176	2.201949
KEGG_PATHWAY	bta05160:Hepatitis C	4	0.00253	*PIK3R3, STAT1, STAT3, STAT2*	13.35692	2.623742

*KEGG, Kyoto Encyclopedia of Genes and Genomes.*

## Discussion

The *STAT* gene family is a family of latent transcription factors that can be activated in response to intracellular stimuli. Over 40 different cytokines or growth factors that can activate the STAT signaling pathway have been found, such as Janus kinases (JAKs), growth factor receptor (EGFR), and the platelet-derived growth factor receptor (PDGFR) (Swiatek-Machado and Kaminska, 2020). The activation of STAT members is temporary (from a few minutes to several hours) under normal physiological conditions. However, constitutive activation of STATs has been identified in multiple cancers, including glioma.

Signal transducer and activator of transcription 1 has been implicated in the pathophysiological processes of several types of cancers and plays dual roles. In colorectal carcinoma, hepatocellular carcinoma, and pancreatic cancer, STAT1 acts as a tumor suppressor. High expression of STAT1 was indicative of a good prognosis and could induce apoptosis or cell cycle arrest (Chen et al., 2013; Gordziel et al., 2013; Sun et al., 2014). However, studies have also shown that high *STAT1* mRNA levels correlated with poor prognosis in breast cancer, while high levels of STAT1 activity promoted cell growth and immune suppression in breast cancer (Tymoszuk et al., 2014; Hou et al., 2018). These findings indicated that the function of STAT1 had a tissue specificity. In glioma, previous studies showed that the expression of STAT1 was negatively correlated with the grade of glioma (Ju et al., 2013; Yang et al., 2018). Overexpression of STAT1 could significantly inhibit cell proliferation and increase cell apoptosis (Zhang et al., 2018). Mechanistically, STAT1 could negatively modulate the expression of mouse double minute 2 (Mdm2) and interact directly with p53 to induce the expression of pro-apoptotic genes, such as *Bax* and *Fas* (Townsend et al., 2004). Suppressors of cytokine signaling 1 (SOCS1), cyclin-dependent kinase inhibitor 1B (Cdkn1b), and VEGFA (Yoshimura, 2009; Zhang et al., 2018) have also been identified as downstream targets of STAT1. However, a more recent study showed that IL-8 promoted glioma migration, invasion, and mesenchymal transition by regulating the STAT1/HIF-1α/Snail axis. Upregulation of *STAT1* signaling genes in GBM was associated with poor prognosis (Duarte et al., 2012; Thota et al., 2014). Furthermore, STAT1 has also been implicated in the immunosuppression of cancer cells. IFN-γ could activate the STAT1/MEK/ERK signaling pathway, and promote the expression of PD-L1 (Liu et al., 2007). In our study, we found that the expression of STAT1 was significantly higher in GBM when compared with LGG in both TCGA and CGGA datasets. STAT1 expression was relatively higher in the *IDH* wild-type group. Paradoxically, high expression of STAT1 predicted a poorer prognosis. This inconsistency between the expression and prognosis of STAT1 requires further research and exploration.

Signal transducer and activator of transcription 2 has been implicated in the process of multiple cancers, such as ovarian cancer (Chen et al., 2020b) and non-small cell lung cancer (Yang et al., 2019). Some studies have shown that STAT2 may be a tumor suppressor by acting downstream of IFN-I (Wang et al., 2003). However, other studies have also shown that STAT2 could promote the tumorigenesis of colorectal cancer (CRC) by upregulating the expression of IL-6 and activating the STAT3 signaling pathway (Gamero et al., 2010). STAT2, along with STAT1 and interferon regulatory factor 9 (IRF9), forms ISGF3 complexes, which play important roles in the activation of immune cells, production of inflammatory cytokines, and immune cell propagation (Lee et al., 2020). In glioma, the expression of STAT2 was lower in LGG than in GBM. In this study, we found that the expression of STAT2 was much higher in the GBM and *IDH* wild-type groups. Higher expression of STAT2 predicted a poorer prognosis. The mechanism and function of STAT2 in glioma needs to be further elucidated.

As a result of dysregulated upstream events and a lack of negative *STAT3* regulation, *STAT3* has been shown to be constitutively activated in glioma. Previous studies indicate that dysregulated *STAT3* promoted cell proliferation, angiogenesis, resistance to apoptosis, and immune escape. Bcl-xL, Bcl2l1, Bcl-2, cyclin D1, and c-Myc have been identified as its downstream targets (Puram et al., 2012). However, recent studies have also shown that the role of STAT3 in glioma is correlated to a degree with genetic alterations. For example, in *PTEN*-deficient GBM (∼35% of GBM), *STAT3* played the role of tumor suppressor rather than of oncogene (Cancer Genome Atlas Research Network, 2008). Aberrant STAT3 signaling in GBM has also been associated with the dysfunction of both the innate and adaptive components of the immune system. The activation of STAT3 in GBM-resident tumor-associated macrophages/microglia (TAMs) impaired their ability to mediate phagocytosis and led to their polarization toward an immunosuppressive phenotype. STAT3 activation inhibited the maturation of dendritic cells and the expression of key molecules necessary for T cell activation and antigen presentation (Cheng et al., 2003). In the adaptive immune system, STAT3 activation promoted an increased proportion of Tregs and decreased infiltration of CD4 + and CD8 + T cells (Zhang et al., 2009).

Signal transducer and activator of transcription 4 has been identified as an oncogene in gastric cancer, ovarian cancer, and CRC (Zhou et al., 2014; Cheng et al., 2015; Zhao et al., 2017). However, high levels of *STAT4* expression predict better prognosis in breast cancer, hepatocellular carcinoma, and gastric cancer (Wang et al., 2015, 2018b). In our study, we found that the expression of *STAT4* had no correlation with tumor grades or patient prognosis.

Signal transducer and activator of transcription 5 signaling has been involved in the tumorigenesis of GBM. STAT5A and STAT5B are predominantly located in the cytoplasm of glioma cells. A study that contained nine normal cortex, 22 diffuse astrocytoma, and 15 GBM samples showed that high levels of STAT5B were detected in 57.1% of GBM samples, 27.3% of diffuse astrocytoma samples, and 22.2% of normal cortex samples. However, high levels of STAT5A were detected in 28.6%, 18.2%, and 22.2% of the GBM samples, diffuse astrocytoma samples, and normal cortex samples, respectively (Liang et al., 2009). Targeting STAT5B, but not STAT5A may suppress GBM cell growth and induce G1 cell cycle arrest. In addition, Bcl-2, p27kip1, p21waf1/cip1, VEGF, and FAK may be downstream targets of STAT5B. Recent studies have shown that STAT5A can promote the transcription of *LINC01198*, which promotes the proliferation of glioma cells by stabilizing DGCR8 (Tan et al., 2020). In addition, STAT5 has been reported to be involved in the maintenance of normal immune function and homeostasis by mediating the biological actions of γc family. STAT5 also plays an important role in the function and development of Tregs. Consistent activation of STAT5 leads to a suppression in antitumor immunity (Rani and Murphy, 2016). In our study, we found that *STAT5A* expression was positively correlated with tumor grade. Higher expression of *STAT5A* indicated poor prognosis, while STAT5B displayed the contrary result. These findings indicated that STAT5A and STAT5B may have opposite roles in glioma.

Studies have shown that *STAT6* was expressed in many glioma tissues but not in normal brain tissue. Upregulation of STAT6 was correlated with longer survival times. A reduction of ^3^H-Thymidine uptake was observed in STAT6-deficient GBM cells. Overexpression of STAT6 promoted cell proliferation and invasion. Urokinase Plasminogen activator (*uPA*) and matrix metalloproteinase 1 (MMP-1) were identified as the target genes of STAT6 (Merk et al., 2011), which have been reported to be expressed by T cells and B cells (Goenka and Kaplan, 2011). STAT6 plays a crucial role in Th2 cell differentiation and promotes the development and differentiation of B cells (Wang et al., 2021). We found that the expression of STAT6 positively correlated with tumor grades and high levels of STAT6 predicted poor prognosis.

In the present study, the association between the *STAT* gene family and glioma was investigated using the TCGA and CGGA databases. We found that the expression of *STAT1*, *STAT2, STAT3, STAT5A*, and *STAT6* was upregulated in GBM compared with LGG tissues, while the expression of *STAT5B* was downregulated. No significant variation in expression was observed when comparing the expression of *STAT4* in both datasets. High expression levels of *STAT1/2/3/5A/6* indicated poorer prognosis, while the expression of *STAT5B* was negatively correlated with prognosis. No apparent correlation between *STAT4* expression and the prognosis of glioma was observed. In addition, subgroup analysis was performed to evaluate the prognostic value of STATs. The results indicated that neither STAT expression nor our constructed model had a significant correlation with prognosis in GBM. This may be due to the median patient survival time of GBM being very short (Perry et al., 2017).

Cancer associated inflammation and immunity can promote the initiation and progression of malignant tumors (Mantovani et al., 2008), and thus, cancer immunotherapy has attracted increasing attention in recent years. Studies have shown that the *STAT* gene family plays an important role in inducing and maintaining cancer-associated inflammation during carcinogenesis and cancer progression (Bollrath et al., 2009; Yu et al., 2009). In this study, we showed that *STAT1/2/3/5A/5B/6* were positively correlated with TIICs, while *STAT4* was negatively associated with TIICs except for CD8 + cells in LGG. GBM is noted for its paucity of T cells and enrichment of macrophages and microglia. The glioma microenvironment of GBM has also been associated with tumor-intrinsic transcriptional subtypes (Cancer Genome Atlas Research Network, 2008; Louis et al., 2016). For example, NF1 deficiency resulted in increased infiltration of tumor-associated macrophages and microglia (Wang et al., 2018a). In this study, we found that CD8 + cells were negatively correlated with almost all STATs. *STAT1/3/5A/5B/6* isoforms were positively correlated or had no significant correlations with other immune cells. The associations between STATs and cancer associated immunity in GBM needs to be further explored in different cell subtypes.

## Conclusion

Our results revealed a dysregulation or disordered expression of the *STAT* gene family in glioma tissues. The prognostic value of *STATs* in glioma was also evaluated and used to construct new prognostic gene signatures with improved predictive value in predicting glioma survival. *STATs* and their co-expressed genes were mainly involved in the following pathways: signal transducer activity, Hepatitis B, *Jak-STAT* signaling pathway, measles, transcription factor activity, and sequence-specific DNA binding. The association between *STATs* and TIICs was also evaluated in LGG and GBM. Further investigation and validation were needed to demonstrate these results in the future.

## Data Availability Statement

The original contributions presented in the study are included in the article/Supplementary Material, further inquiries can be directed to the corresponding author/s.

## Author Contributions

WJ, YL, and BX designed the study and analyzed the data. JM, CC, and WH checked the data. YX and KY reviewed the process. JJ, HL, and JS wrote and revised the manuscript. All authors have read and approved the final manuscript.

## Conflict of Interest

The authors declare that the research was conducted in the absence of any commercial or financial relationships that could be construed as a potential conflict of interest.

## Footnotes

1 http://www.oncomine.org/

2 http://cancergenome.nih.gov/

3 http://www.cgga.org.cn/

4 www.proteinatlas.org

5 http://www.cbioportal.org/

6 https://cistrome.shinyapps.io/timer/

## Abbreviations

STAT: signal transducer and activator of transcription
TCGA: The Cancer Genome Atlas
CGGA: Chinese Glioma Genome Atlas
ROC: receiver operating characteristic
TIICs: tumor-immune infiltrating cells
TIMER: tumor immune estimation resource
PPI: protein-protein interaction
GO: gene ontology
KEGG: Kyoto Encyclopedia of Genes and Genomes
BP: biological processes
CC: cellular component
MF: molecular functions
*JAKs*: Janus kinases
LGG: low grade glioma.
