# Supplementary material for: Bioinformatics Analysis of Expression Profiles and Prognostic Values of the Signal Transducer and Activator of Transcription Family Genes in Glioma
Source: Front Genet. 2021 Jul 2;12:625234. doi: 10.3389/fgene.2021.625234 (PMC8283826; doi:10.3389/fgene.2021.625234)
Supplement: Supplementary file 1 [file Data_Sheet_1.docx]

**Supplemental material Table1. The association between STATs and clinicopathological characteristics in TCGA**

| Characteristics | n | STAT1 | | P Value^a^ | STAT2 | | P Value^a^ | STAT3 | | P Value^a^ | STAT4 | | P Value^a^ | STAT5A | | P Value^a^ | STAT5B | | P Value^a^ | STAT6 | | P Value^a^ | The new model | | P Value^a^ |
| --- | --- | --- | --- | --- | --- | --- | --- | --- | --- | --- | --- | --- | --- | --- | --- | --- | --- | --- | --- | --- | --- | --- | --- | --- | --- |
|  |  | Low | High |  | Low | High |  | Low | High |  | Low | High |  | Low | High |  | Low | High |  | Low | High |  | Low | High |  |
| Age |  |  |  |  |  |  |  |  |  |  |  |  |  |  |  |  |  |  |  |  |  |  |  |  |  |
| <60 | 504 | 324 | 180 | <0.0001 | 299 | 205 | <0.0001 | 276 | 228 | 0.0012 | 295 | 209 | 0.0021 | 250 | 254 | <0.0001 | 201 | 303 | <0.0001 | 274 | 230 | <0.0001 | 322 | 182 | <0.0001 |
| ≥60 | 152 | 55 | 97 |  | 61 | 91 |  | 60 | 92 |  | 67 | 85 |  | 46 | 106 |  | 97 | 55 |  | 55 | 97 |  | 37 | 115 |  |
| Gender |  |  |  |  |  |  |  |  |  |  |  |  |  |  |  |  |  |  |  |  |  |  |  |  |  |
| Female | 282 | 170 | 112 | 0.2648 | 166 | 116 | 0.0814 | 149 | 133 | 0.4788 | 157 | 125 | 0.8740 | 135 | 147 | 0.2349 | 120 | 162 | 0.2060 | 147 | 135 | 0.3868 | 167 | 115 | 0.0478 |
| male | 374 | 209 | 165 |  | 194 | 180 |  | 187 | 187 |  | 205 | 169 |  | 161 | 213 |  | 178 | 196 |  | 182 | 192 |  | 192 | 182 |  |
| WHO grades |  |  |  |  |  |  |  |  |  |  |  |  |  |  |  |  |  |  |  |  |  |  |  |  |  |
| LGG | 509 | 350 | 159 | <0.0001 | 312 | 197 | <0.0001 | 302 | 207 | <0.0001 | 286 | 223 | 0.3476 | 263 | 246 | <0.0001 | 174 | 335 | <0.0001 | 292 | 217 | <0.0001 | 350 | 159 | <0.0001 |
| GBM | 147 | 29 | 118 |  | 48 | 99 |  | 34 | 113 |  | 76 | 71 |  | 33 | 114 |  | 124 | 23 |  | 37 | 110 |  | 9 | 138 |  |
| IDH type |  |  |  |  |  |  |  |  |  |  |  |  |  |  |  |  |  |  |  |  |  |  |  |  |  |
| Wild type | 254 | 75 | 179 | <0.0001 | 101 | 153 | <0.0001 | 75 | 179 | <0.0001 | 114 | 140 | <0.0001 | 68 | 186 | <0.0001 | 180 | 74 | <0.0001 | 76 | 178 | <0.0001 | 46 | 208 | <0.0001 |
| Mut type | 402 | 304 | 98 |  | 259 | 143 |  | 261 | 141 |  | 248 | 154 |  | 228 | 174 |  | 118 | 284 |  | 253 | 149 |  | 313 | 89 |  |

**Supplemental material Table2. The association between STATs and clinicopathological characteristics in CGGA**

| Characteristics | n | STAT1 | | P Value^a^ | STAT2 | | P Value^a^ | STAT3 | | P Value^a^ | STAT4 | | P Value^a^ | STAT5A | | P Value^a^ | STAT5B | | P Value^a^ | STAT6 | | P Value^a^ |
| --- | --- | --- | --- | --- | --- | --- | --- | --- | --- | --- | --- | --- | --- | --- | --- | --- | --- | --- | --- | --- | --- | --- |
|  |  | Low | High |  | Low | High |  | Low | High |  | Low | High |  | Low | High |  | Low | High |  | Low | High |  |
| Age |  |  |  |  |  |  |  |  |  |  |  |  |  |  |  |  |  |  |  |  |  |  |
| <60 | 196 | 139 | 57 | 0.0159 | 121 | 75 | 0.0659 | 121 | 75 | 0.3020 | 147 | 49 | 0.6498 | 123 | 73 | 0.0034 | 102 | 94 | 0.0678 | 137 | 59 | 0.0024 |
| ≥60 | 28 | 13 | 15 |  | 12 | 16 |  | 14 | 14 |  | 20 | 8 |  | 9 | 19 |  | 20 | 8 |  | 11 | 17 |  |
| Gender |  |  |  |  |  |  |  |  |  |  |  |  |  |  |  |  |  |  |  |  |  |  |
| Female | 84 | 59 | 25 | 0.6578 | 57 | 27 | 0.0500 | 54 | 30 | 0.3980 | 64 | 20 | 0.7519 | 51 | 33 | 0.7792 | 42 | 42 | 0.3332 | 58 | 26 | 0.5601 |
| male | 140 | 93 | 47 |  | 76 | 64 |  | 81 | 59 |  | 103 | 37 |  | 81 | 59 |  | 80 | 60 |  | 90 | 50 |  |
| WHO grades |  |  |  |  |  |  |  |  |  |  |  |  |  |  |  |  |  |  |  |  |  |  |
| LGG | 139 | 109 | 30 | <0.0001 | 103 | 36 | <0.0001 | 104 | 35 | <0.0001 | 105 | 34 | 0.7522 | 101 | 38 | <0.0001 | 69 | 70 | 0.0730 | 114 | 25 | <0.0001 |
| GBM | 85 | 43 | 42 |  | 30 | 55 |  | 31 | 54 |  | 62 | 23 |  | 31 | 54 |  | 53 | 32 |  | 34 | 51 |  |
| IDH type |  |  |  |  |  |  |  |  |  |  |  |  |  |  |  |  |  |  |  |  |  |  |
| Wild type | 109 | 56 | 53 | <0.0001 | 43 | 66 | <0.0001 | 43 | 66 | <0.0001 | 77 | 32 | 0.2206 | 42 | 67 | <0.0001 | 72 | 37 | 0.0008 | 42 | 67 | <0.0001 |
| Mut type | 115 | 96 | 19 |  | 90 | 25 |  | 92 | 23 |  | 90 | 25 |  | 90 | 25 |  | 50 | 65 |  | 106 | 9 |  |

^a^*p* value for χ^2^ test
